# AZAR eye and vision cohort study

**DOI:** 10.1038/s41598-023-30212-y

**Published:** 2023-05-17

**Authors:** Mohammad Hossein Somi, Zeinab Nikniaz, Alireza Ostadrahimi, Seyed Sina Naghibi Irvani, Amir Mohammad Nourizadeh, Mohammad Mirzaei, Fateme Alipour, Fatemeh Jafari, Elnaz Faramarzi

**Affiliations:** 1 Liver and Gastrointestinal Diseases Research Center of Tabriz University of Medical Sciences, P.O. Box: 1567812907, Tabriz, Iran; 2 Nutrition Research Center, Tabriz University of Medical Sciences, Tabriz, Iran; 3 Department of Ophthalmology, Tabriz University of Medical Sciences, Tabriz, Iran; 4 Translational Ophthalmology Research Center, Tehran University of Medical Sciences, Tehran, Iran; 5 Eye Research Center, Farabi Eye Hospital, Tehran University of Medical Sciences, Tehran, Iran

**Keywords:** Eye diseases, Risk factors, Epidemiology, Ecological epidemiology, Climate-change impacts

## Abstract

According to World Health Organization (WHO), currently, 2.2 billion people are living with visual impairment worldwide, of which almost half could have been prevented. There are both modifiable and unmodifiable factors leading to visual disability and, ultimately, blindness. Several population-based studies in different parts of Iran have tried to determine these factors concerning their specific population and environment-related characteristics. AZAR Eye and Vision cohort is the second-largest cohort study in the whole country. AZAR Eye and Vision cohort is the ophthalmologic branch of AZAR cohort which is the largest eye cohort study in the country, which is trying to determine the prevalence and incidence of visual impairment, blindness, and other major ophthalmologic conditions and their associated risk factors in East Azerbaijan province located in Iran, a middle eastern country. A recently emerging phenomenon is the drying of the ultra-salty lake of Urmia located in the West Azerbaijan province which is a direct neighbor of our studied population and has caused recurrent salt storms in the immediate near areas. This phenomenon could adversely affect visual health via different conditions which our study will elucidate. The enrollment phase took place between 2014 and 2017 and 11,208 participants were enrolled out of 15,000 participants in the primary cohort. The resurvey phase will begin five years after the enrollment phase. In this phase, 30% of the participants are randomly selected to be reexamined and complete questionnaires. The participants showing any issues such as diabetes and being a glaucoma suspect will be included in the resurvey phase, too. Data categories gathered include demographics, lifestyle factors, past medical and drug histories, and a diet quality and quantity questionnaire including 130 edible items. Urine, hair, nail, and 25-ml blood samples, were collected from the participants. Then they were referred to an optometrist to complete an ophthalmologic questionnaire and undergo eye examination and lensometry. Then they underwent slit-lamp examinations and pictures were taken of the lens and fundus. People with suspected visual impairment were referred to an ophthalmology clinic. The data are processed and a four-level quality check is performed on each block. The most common visual impairment is cataracts. This study’s most important aim is to evaluate the effect of local environmental and ethnic factors on eye diseases in this specific population.

## What was the purpose of the cohort?

Visual impairment is a global health issue that is linked to a variety of functional and mental comorbidities. According to the World Health Organization, almost 2.2 billion people worldwide have vision impairment, with 36 million blind^1^. Visual abnormalities are preventable and treatable in about 80% of cases. As a result, several studies have been done to discover modifiable factors related to visual impairment^2^, and various preventative programs have been established based on these findings to prevent this issue. However, previous research has found that the prevalence of vision impairment and blindness is heavily influenced by ethnicity and environmental factors. As a result, determining the size and epidemiology of visual impairments and their risk factors in each location is critical for developing appropriate and successful strategies to address this problem.

In this context, a small number of population-based ocular studies in different parts of Iran attempt to answer this topic. The "Tehran eye study" is one of Iran's most significant eye studies, done in Tehran province [the country's capital city] with no follow-up phases^3^. However, prospective cohort studies are needed to better demonstrate the causation relationship for vision impairment and examine the gene-environment interaction. The Shahroud eye cohort was done on a 40–64-year-old population in North East Iran to determine the prevalence of eye illnesses and their prognosis and determinants^4^. Given that Iran is home to a diverse range of ethnic groups and distinct environmental risk factors in different parts of the country, it appears necessary to investigate genetic and environmental risk factors in each location. In this context, the Persian eye cohort study and the Persian cohort study in several regions of Iran, including the northwest, began in 2014. East Azerbaijan, located in Iran's northwestern corner, has recently experienced an environmental disaster in Lake Urmia (one of the largest salt lakes). It's been suggested that the Urmia lake drying in this area is linked to various health issues. There is a rare study regarding the effect of this disaster on the health status of the population in this region. The pilot study results indicated that Urmia lake drying provokes hypertension and anemia^5^. Hypertension increases the risk of some eye diseases such as cataracts^6^ and glaucoma^7^.

Moreover, there are reports that salt dust storms contain potentially toxic components which could threaten human health. According to the suggestion of all studies in this field, conducting well-designed prospective studies are necessary^5,8,9^. Therefore, the AZAR Eye and Vision cohort study aimed to determine environmental risk factors for eye diseases by comprehensively assessing external exposures, lifestyle (including physical activity, and dietary patterns), social determinants, and ethnic and individual characteristics; and to design population-based prospective studies of causal disease mechanisms by using the advantage of natural changes in regions of rapid environmental and social transition.

## Who is a member of the cohort?

The AZAR cohort is a long-term cohort study of non-communicable diseases and associated risk factors in the Shabestar county of East Azarbaijan province, near the Urmia Lake in Iran's northwest^10^. The research included an epidemiologic investigation of eye disease among residents aged 35 to 70, which started in 2014 and concluded in 2017.

The AZAR cohort is a large population-based cohort that is part of the PERSIAN Cohort Study (Prospective Epidemiological Research Studies in Iran, ethics number: IR.TUMS.DDRI.REC.1396.1)^11^. The Ethical Committee of Tabriz University of Medical Sciences has authorized the AZAR cohort (record number: tbmed.rec.1393.205). We hereby confirm that all methods were performed in accordance with the with the declaration of Helsinki. All participants gave their written informed consent, and they were free to exit the study at any time and for any reason. The AZAR cohort population was detailed in further depth in the published cohort profile. Inclusion and exclusion criteria for AZAR Eye and Vision cohort study, are exactly the same as AZAR cohort study, previously published. The inclusion criteria was: (1) permanent or a minimum of 9 months residence each year in Shabestar district; (2) written informed consent; (3) at least one parent of Azeri ethnicity; and (4) age between 35 and 70 years at the time of enrollment in the study. Exclusion criteria was limited to having a disabling psychiatric condition or motor disability, which would limit effective cooperation with the interviewer or the examining physician.

The aims of the AZAR Eye and Vision cohort study were described to patients who participated in the AZAR Eye and Vision cohort study. When they agreed to participate in the AZAR Eye and Vision cohort, they were given an eye examination in addition to the AZAR cohort study protocols. Between October 2014 and June 2017, 11,028 people from the AZAR cohort took part in the AZAR Eye and Vision cohort study, out of 15,000 participants in the primary cohort. After six months, an optometrist and an ophthalmologist reexamined all individuals with diabetes (self-reported or newly confirmed diabetes) of 11,028 subjects.se

## How often have they been followed up?

The study's enrollment phase was finished in February 2017. The study's resurvey phase was expected to begin five years following the enrolment phase but has been delayed due to the COVID-19 worldwide pandemic. In the interval between enrollment and resurvey phase completion, all participants are contacted by phone once a year. They've been questioned about all of their health-related problems including eye related problems (any newly diagnosed eye disease or any systemic diseases affecting the eyes, newly performed eye surgeries, ophthalmologist visits of any kind or any eye related hospital admissions) and finally they have been consulted on whether or not they should be referred to an ophthalmologist. One of the main purposes of these annual phone follow-ups between the enrollment and resurvey phase and the targeted questions surveyed during these contacts were to better assess for any possible detrimental effects of the drying of the Lake Urmia, on eye and visual health of the residents. As the drying of an ultra-salty lake is an extremely rare ecologic event in a human habited area, throughout the history, it makes this cohort very unique in its ability to register any possible correlation between eye diseases and this change; hence we have designed the study in a manner to minimize the possibility of missing any cause-and-effect relationship.

In the resurvey phase, thirty percent of the enrollment phase population will be randomly selected for reexamination by optometrists and ophthalmologists and completion of all enrollment phase questionnaires. All participants who had any concerning issue found in phase 1 (including diabetes, abnormal fundus or slit photo, any eye disease resulting in visual acuity < 7/10, being glaucoma suspect) will be followed up regardless of being included in the randomly selected 30% or not.

## What has been measured?

Because this study is part of the AZAR cohort study, a large amount of information was gathered during the enrolling phase. The interviews were conducted using a block design, elaborated upon in great detail in prior published articles^10^. The following is a summary of the data gathered:

The most common data categories include demographics, socioeconomic status, lifestyle factors (such as physical activity and substance use), occupational history (including occupational exposures), past medical history, medicine use (past and present), family medical history including family history of eye diseases, gynecological and obstetrics history (for women), oral and dental health, circadian rhythm, and dietary habits (including food processing, water supply, housing, cellphone use, pesticide exposure). A food frequency questionnaire is employed to evaluate dietary consumption, consisting of 130 items such as bread and cereals, meats, dairy products, oils, sweets, legumes, vegetables, fruits, and sauces. Height, weight, waist, hip, wrist circumferences, and blood pressure were all measured (systolic and diastolic). In this study, participants' socioeconomic status was evaluated as the Wealth Score Index (WSI). Based on possession of a range of durable assets (for example, dishwasher, car, and TV), house condition (e.g., the number of rooms, type of ownership), and educational levels, WSI was calculated by Multiple Correspondence Analysis (MCA). WSI was classified into five WSI quintiles, ranging from the lowest to the highest ones (1st to 5th quintile, respectively).

### Biological specimen

After an overnight fast of 12–14 h, 25-ml blood samples are drawn from peripheral veins, and serum, plasma, and buffy coat are separated. For future biochemical testing, the materials are kept at − 80 °C in two-dimensional barcoded aliquots. Following sample collection, biochemical parameters such as serum fasting blood glucose (FBS), complete blood count (CBC), blood urea nitrogen (BUN), creatinine, lipid profiles (total cholesterol, low- and high-density lipoprotein, triglyceride), and liver enzymes (aspartate, alanine, and gamma-glutamyl aminotransferase) are measured. Urine tests are performed. Urine, hair, and nail samples are collected and stored.

### Section on ophthalmology

Participants were referred to an optometrist after completing all modules. The optometrist initially filled up a questionnaire for each individual, which consisted of seven questions and sixteen items. Information about the participants' medical conditions, such as diabetes history, family history of eye diseases like glaucoma, retinal detachment, keratoconus, and retinitis pigmentosa (RP) were noted in this questionnaire. In addition, the next section of the questionnaire asks about glasses/contact lens wearing, dry eye symptoms, amblyopia treatment, eye surgeries or procedures (such as cataract surgery, any type of laser photocoagulation therapy (including Pan-Retinal Photocoagulation [PRP]) for Proliferative Diabetic Retinopathy (PDR), corrective surgeries for refractive eye errors, glaucoma surgery, etc.), eye care and optometry appointments. The optometrist conducted an eye examination once the questionnaire was completed.

### Examinations by an optometrist

During the trial, a single trained optometrist examined the participants' eyes. Lensometry of present glasses, measuring distance visual acuity, determining objective and subjective refraction, assessing eye movement, measuring IOP (intraocular pressure), and gross examination of lid lesions were all part of an optometry examination.

### Lensometry

The non-automatic analog Topcon lensometer was used to assess the participants' spectacles if any were present (Topcon Corporation, Tokyo, Japan).

### Visual acuity

The Snellen chart projector (CP-770, NIDEK, Japan) was used to determine uncorrected visual acuity (VA). As per the Persian cohort methodology, the right eye was measured first (submitted article|). The testing was done at a 6-m distance. The vision testing began at the top of the chart and proceeded until more than half of the letters were misread or the patient read all of the letters.

### Refraction

The NIDEK Autorefractometer (AR1, NIDEK, and Japan) was initially used to assess non-cycloplegic autorefraction in the right eye. Reports of the sphere, cylinder, and axis were recorded automatically for each eye. In this section, the poor red reflex (PRP) and scissor motion (SM) of each participant were evaluated by optometrists.

### Relative afferent pupillary defect test (RAPD)

The presence of Relative afferent Pupillary Defect (RAPD) in the AZAR Eye and Vision cohort population was checked using the swinging light test. This method was described briefly here and was explained in more detail in the other article (in press). After dimming the room's lighting, the light source from a direct ophthalmoscope (BETA ®200, HEINE, Germany) was held on each pupil for at least 3 s. Based on the simultaneous dilatation of both pupils, RAPD was defined.

### Intraocular pressure

Goldman applanation tonometry (OPTILASA, Spain) was used to determine IOP. Each eye was measured once, with the right eye being measured first.

### Other examination

Corneal opacities and eyelid lesions, such as ptosis, entropion, ectropion, etc., were evaluated by slit-lamp (SL-3G, TOPOCON, Japan).

### Ocular photography

We took slit-lamp pictures of the lens and fundus for this study. A Topcon SL-3G Photo slit lamp (SL-3G, TOPOCON, Japan) was used for slit-lamp and lens photography. Slit-lamp photography is a photograph of the eye, including the lid margins, cornea, and iris. Dilation was used to photograph the lenses. The nucleus focuses on one photo, while the lens is retro illumination in the other. For fundus photography, we used a Digital Retinal Camera (CR2-AF Canon Japan) according to the Early Treatment Diabetic Retinopathy Study (ETDRS). In nondiabetic individuals, we took three conventional pictures from the first three ETDRS fields and one stereoscopic image from the optic nerve in each eye. Both eyes of diabetic patients were photographed using the ETDRS seven-standard field method. After maximum dilation of the pupils with mydrax 1% to each eye, two pictures were obtained using a digital fundus camera (CR2-AF Canon Japan) coupled with a 90-diopter lens camera (Volk Digital Wide Field ®, USA) according to the cohort protocol. At a 45° angle, all patients had two-field macula-centered fundus imaging. Participants were separated into three groups once the optometrists completed all necessary steps: normal, a requirement for spectacles, or referral to an ophthalmologist.

### Criteria for referral

A comprehensive ophthalmic examination was performed on all who were referred. Diabetic, family history of glaucoma, IOP > 20 mmHg, Relative afferent pupillary deficit (RADP+), best-corrected visual acuity (BCVA) < 8/10, poor red reflex, moderate to severe dry eye symptoms, suspicion of scissor reflex, eyelid abnormalities and any other suspicious findings were among the criteria used by the optometrist to refer patients to an ophthalmologist.

### Ophthalmologic examination

Those who were referred to an ophthalmologist underwent a thorough examination. First and foremost, the ophthalmologist examined the client's eyes with the naked eye. Examination of the eyelid (entropion, ectropion), lacrimal system, eyelid bulk, dry eye, extraocular muscles, and conjunctiva were among the items investigated. Slit-lamp biomicroscopy and intraocular pressure measurements were performed after these examinations. Dilatation of the pupils after pupil dilatation, clinical lens opacity grading, and vitreous examination was performed. The slit lamp was used to assess opacities, and direct and indirect ophthalmoscopy was used to examine the fundus. We employed an SL-3G, TOPOCON for slit-lamp (SL-3G, TOPCON, Japan).

After dilatation, lens opacity grading was performed at the slit lamp in addition to lens photography. The ophthalmologist used standard images from the WHO simplified cataract grading system to make comparisons. The ophthalmologist examined the retina and optic disc thoroughly and systematically with direct and indirect ophthalmoscopy, recording all findings.

Participants with resolvable eye issues were treated with the guidance and treatment of an ophthalmologist, or a treatment and follow-up program was established for them after a thorough ophthalmological examination.

People with suspected lesions, such as glaucoma or retinal disorders, were referred to an ophthalmology referral center for further diagnostic and treatment measures and counseling.

### Quality control and data processing

A four-level quality check is performed on each block. A basic review is done by the software so that appropriate data are recorded in each part in terms of variable kinds, length, and measurement levels. The program conducts a basic check to verify that the correct data is captured in each questionnaire part. When information is missing, the operator is prompted to fill in the gaps. Furthermore, during the first phase, an independent staff member conducts frequent quality monitoring, which is monitored using a validated checklist. General inquiries, anthropometric measurements, medical and disease screening questionnaires, and biological sample collection and biobank maintenance are all examined by the team for each participant. If any information is missing or incomplete, the record will be indexed and sent to another team to finish. It is possible to import just verified entries into the database. An epidemiologist next evaluates the records. The quality control administrator, who is an AZAR principal investigator with full access to the whole records database, verifies the data at random. Moreover, the Persian cohort team performs central quality checks on quantitative data.

## What has it found?

**Figure 1:**
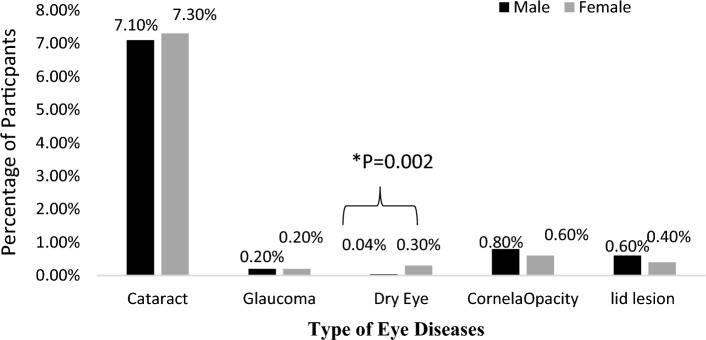
Prevalence of different types of eye diseases in Azar cohort population stratified by gender. *P: Chi-Square test.

**Figure 2:**
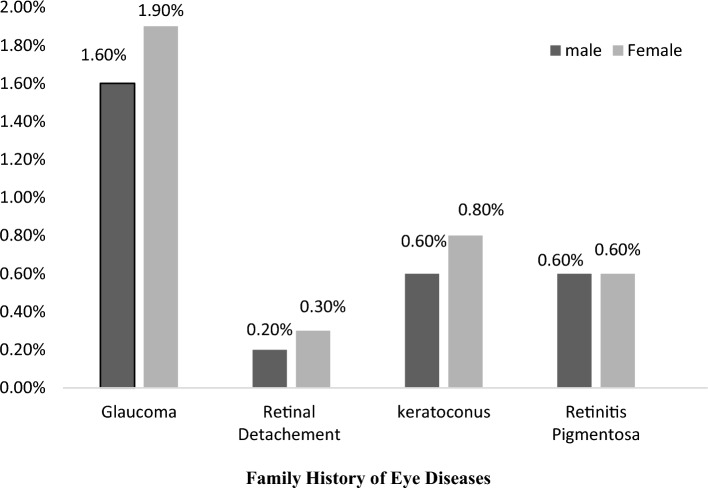
Prevalence of positive family history for different types of eye diseases in Azar cohort population stratified by gender.

The baseline characteristics of the participant in the AZAR Eye and Vision cohort are presented in Table 1. As shown in Fig. 1, the most prevalent eye disease in males and females, at baseline was cataract 7.1% and 7.3% respectively. In Fig. 1, we observed that the prevalence of dry eye in females was significantly (*P* = 0.002) higher than males (0.3% vs. 0.04%). In both genders, the most prevalent family history of eye diseases was glaucoma (Fig. 2). Only 1% of the population has IOP > 21 mmHg (Table 2).

**Table 1:** Demographics characteristics of Azar cohort population.

	Male (n = 4992)		Female (n = 6216)		*P	Total (n = 11,208)	
	n	%	N	%		n	%
Age (years) classification					< 0.001		
35–45	1678	33.6	2364	38		4042	36.1
46–55	1681	33.7	2103	33.8		3784	33.8
56–65	1295	25.9	1400	22.5		2695	24
66–70	338	6.8	349	5.6		687	6.1
Marital status					< 0.001		
No Married	56	1.1	748	12		804	7.2
Married	4936	98.9	5468	88		10,404	92.8
Residential regions					0.03		
Urban residents	3110	62.3	3768	60.6		6878	61.4
Rural residents	1882	37.7	2448	39.4		4330	38.6
Education level					< 0.001		
Illiterate	464	9.3	1496	24.1		1960	17.5
Primary school	1915	38.4	2653	42.7		4568	40.8
Diploma	2074	41.5	1785	28.7		3859	34.4
University	539	10.8	282	4.5		821	7.3
Quintiles of wealth index					< 0.001		
1 (poorest)	887	17.8	1744	28.1		2631	23.5
2	816	16.3	1115	17.9		1931	17.2
3	1107	22.2	1246	20		2353	21
4	1045	20.9	1271	20.4		2316	20.7
5 (richest )	1137	22.8	840	13.5		1977	17.6
Current Smoking status							
Smoker	1456	29.2	23	0.4	<0.001	1479	13.2
No smoker	3536	70.8	6193	99.6		9729	86.3
BMI (kg/m^2^) classification					< 0.001		
Under weight (< 18.5)	48	1	19	0.3		67	0.6
Normal weight (18.5–24.9)	1370	27.4	938	15.1		2308	20. 6
Over weight (25–29.9)	2276	45.6	2319	37.3		4595	41
Obese (≥ 30)	1298	26	2940	47.3		4238	37.8
Diabetes	605	12.1	922	14.8	< 0.001	1527	13.6
Hypertension	790	15.8	1642	26.4	< 0.001	2432	21.7

*P, Chi-square test.

**Table 2:** Ocular characteristics of eligible participants in the study.

	Male		Female		^¶^P	Total	
	N	%	N	%		n	%
Ocular hypertension mmHg					0.13		
Normal ≤ 21	4932	98.8	6156	99.1		11,088	98.9
High > 21	58	1.2	58	0.9		116	1
Near glass	1372	27.5	1870	30.1	< 0.001	3242	28.9
Far glass	1164	23.3	1267	20.4	0.001	2431	21.7
History of surgery							
Laser diabetic	34	0.7	41	0.7	0.49	75	0.7
Refractive error	32	0.6	34	0.5	0.30	66	0.6
Cataract	276	5.5	331	5.3	0.33	607	5.4
Glaucoma	6	0.1	7	0.1	0.56	13	0.1
Retinal detachment	10	0.2	7	0.1	0.17	17	0.2
Lacrimal duct	16	0.3	62	1	< 0.001	78	0.7
Ophthalmologist visit within past year					< 0.001		
No	3830	76.7	4545	73.1		8375	74.7
Eye diseases	278	5.6	445	7.2		723	6.5
Checkup	884	17.7	1226	19.7		2110	18.8
Referred to ophthalmologic	1101	40.95	1587	59.04		2688	23.9
Reasons for referred to ophthalmologist							
Diabetes	529	42.1	729	57.9	0.14	1258	46.8
Glaucoma or suspected glaucoma	10	38.5	16	61.5	0.48	26	1
*IOP > 20	57	49.1	59	50.9	0.04	116	4.3
**RAPD	22	52.4	20	47.6	0.08	42	1.6
Visual acuity < 8/10	668	37.9	1094	62.1	< 0.001	1762	65.6
Eye aberration	21	45.7	25	54.3	0.3	46	1.7
suspicion of scissor reflex	45	37.8	74	62.2	0.26	119	4.4
Eyelid abnormalities	25	48.1	27	51.9	0.18	52	1.9
Moderate to severe dry eye	3	23.1	10	76.9	0.15	13	0.5
Poor red reflex	39	52.7	35	47.3	0.02	74	2.8

P, chi-Square test.

*Iop, intraocular pressure; **RAPD, Relative afferent pupillary deficit.

The frequency of using near and far glasses in females was significantly (*P* < 0.0001) higher than in males. Of 11,028 subjects, 2688 (23.9%) people were referred to an ophthalmologist by the optometrist.

The main reasons for referring to an ophthalmologist were BCVA < 8/10 (65.6%), diabetes (46.8%), suspicion of scissor reflex (4.4%), and IOP > 20 (4.6%).

## What are the main strengths and weaknesses?

The AZAR Eye and Vision cohort study's key strength is the study's novel concept: to evaluate how a significant environmental change (natural changes at Lake Urmia) affects eye illnesses against a relatively steady genetic predisposition at a population level. The AZAR Eye and Vision cohort studies a population in a specific place with exact environmental change timing. As a result, it avoids several other studies' flaws. The ethnically homogeneous group of the AZAR Eye and Vision cohort will likely provide a higher capacity to test associations. AZAR Eye and Vision cohort is also the first and most extensive study to focus on people of Turkish descent. Another benefit of the experiment is a large amount of data obtained, which will aid in understanding ocular epidemiology and the possibility of investigating many possible correlations. Many procedures were used to ensure the quality of the data acquired. The study's drawback is that our sample size is limited to those aged 35 to 70; thus, issues at younger and older ages cannot be investigated.

## Can I get ahold of the data? Where can I find out more?

The AZAR Eye and Vision cohort collaborates with foreign professionals to further strengthen national and international collaborations. It employs online communication methods, such as its website, to communicate information from the AZAR Eye and Vision cohort, raise project awareness, and attract international collaborators [http://azarcohort.tbzmed.ac.ir].

### Ethics approval and consent to participate

This study was approved by the ethics committee of Tabriz University of medical sciences (tbzmed.rec.1393.205).

## Data Availability

The data that support the findings of this study are available from [Vice Chancellor for Research] but restrictions apply to the availability of these data, which were used under license for the current study, and so are not publicly available. Data are however available from the authors upon reasonable request and with permission of [Vice Chancellor for Research].
